# An Empirical Study on Diabetes Depression over Distress Evaluation Using Diagnosis Statistical Manual and Chi-Square Method

**DOI:** 10.3390/ijerph18073755

**Published:** 2021-04-03

**Authors:** Sohail M. Noman, Jehangir Arshad, Muhammad Zeeshan, Ateeq Ur Rehman, Amir Haider, Shahzada Khurram, Omar Cheikhrouhou, Habib Hamam, Muhammad Shafiq

**Affiliations:** 1 Department of Cell Biology and Genetics, Shantou University Medical College, Shantou 515041, China; mn.sohail@stumail.ysu.edu.cn; 2 Department of Electrical & Computer Engineering, COMSATS University Islamabad, Lahore Campus, Lahore 54000, Pakistan; jehangirarshad@cuilahore.edu.pk; 3 Department of Medicine and Surgery, Al-Nafees Medical College and Hospital, Isra University, Islamabad 44000, Pakistan; mn9892639@gmail.com; 4 Department of Electrical Engineering, Government College University, Lahore 54000, Pakistan; ateeq.rehman@gcu.edu.pk; 5 Department of Intelligent Mechatronics Engineering, Sejong University, Seoul 05006, Korea; amirhaider@sejong.ac.kr; 6 Faculty of Computing, The Islamia University of Bahawalpur, Bahawalpur 63100, Pakistan; Khurram@iub.edu.pk; 7 College of CIT, Taif University, P.O. Box 11099, Taif 21944, Saudi Arabia; o.cheikhrouhou@tu.edu.sa; 8 Faculty of Engineering, Moncton University, Moncton, NB E1A3E9, Canada; Habib.Hamam@umoncton.ca; 9 Department of Information and Communication Engineering, Yeungnam University, Gyeongsan 38541, Korea

**Keywords:** type 2 diabetes mellitus forecasting, diabetes distress, depression, prevalence, clinical implications, regression correlation factors

## Abstract

Diabetes distress is an alternative disorder that is often associated with depression syndromes. Psychosocial distress is an alternative disorder that acts as a resistance to diabetes self-care management and compromises diabetes control. Yet, in Nigeria, the focus of healthcare centers is largely inclined toward the medical aspect of diabetes that neglects psychosocial care. In this retrospective study, specific distress was measured by the Diabetes Distress Screening (DDS) scale, and depression was analyzed by the Beck Depression Inventory (BDI) and Diagnosis Statistics Manual (DSM) criteria in type 2 diabetes mellitus (T2DM) patients of Northwestern Nigeria. Additionally, we applied the Chi-square test and linear regression to measure the forecast prevalence ratio and evaluate the link between the respective factors that further determine the odd ratios and coefficient correlations in five nonintrusive variables, namely age, gender, physical exercise, diabetes history, and smoking. In total, 712 sample patients were taken, with 51.68% male and 47.31% female patients. The mean age and body mass index (BMI) was 48.6 years ± 12.8 and 45.6 years ± 8.3. Based on the BDI prediction, 90.15% of patients were found depressed according to the DSM parameters, and depression prevalence was recorded around 22.06%. Overall, 88.20% of patients had DDS-dependent diabetes-specific distress with a prevalence ratio of 24.08%, of whom 45.86% were moderate and 54.14% serious. In sharp contrast, emotion-related distress of 28.96% was found compared to interpersonal (23.61%), followed by physician (16.42%) and regimen (13.21%) distress. The BDI-based matching of depression signs was also statistically significant with *p* < 0.001 in severe distress patients. However, 10.11% of patients were considered not to be depressed by DSM guidelines. The statistical evidence indicates that depression and distress are closely correlated with age, sex, diabetes history, physical exercise, and smoking influences. The facts and findings in this work show that emotional distress was found more prevalent. This study is significant because it considered several sociocultural and religious differences between Nigeria and large, undeveloped, populated countries with low socioeconomic status and excessive epidemiological risk. Finally, it is important for the clinical implications of T2DM patients on their initial screenings.

## 1. Introduction

Diabetes mellitus (DM) is an exponentially evolving disease of the 21st century around the globe, and its prevention and cure have emerged as a bigger challenge for medical professionals, the pharmaceutical industry, and also the public. A total of 385 million individuals had diabetes in 2013 [1] that may escalate if neglected and even lead to death. According to a survey study of the International Diabetes Federation (IDF) [2], around 483 million people were diagnosed with diabetes in 2019, globally, out of which 326 million people suffer from diabetes in developed countries specifically. African countries share 5.36% of the total number. In sub-Saharan Africa (SSA), the undiagnosed diabetic ratio was estimated at around 93% (out of which 8.92% were men and 9.38% were women, only in Nigeria). According to the World Health Organization (WHO) survey study report of 2019 [3] on the African region, 0.34 million casualties occurred because of diabetes, 80% of which involved people under the age of 60, the most in any region around the globe. Moreover, the American Diabetes Association (ADA) has an estimation of Nigeria regarding the prevalence in both men and women as 21.23% [4]. Further, in the global population correlation, a study conducted by Nigerian Health Organizations (NHO) [5] noted that the prevalence of diabetes in 2010 was 4.7% and would be further projected to 9.3% by 2040 and can even exceed 10% by 2050 [6].

Diabetes mellitus patients encounter numerous difficulties in the treatment of their condition [7,8]. Habits such as smoking and alcohol consumption in large amounts cause distress, anxiety, and depression in patients with diabetes [9,10]. Several works in the literature have been found on diabetic patients outlining the challenges of diabetes management [11,12,13]; however, to the best of our knowledge, the emotional, demographic, and sociographic factors of depression and distress linked to diabetic patients were not examined properly in the existing literature. It has been well understood that there is a strong, obvious correlation present between diabetes and depression [14,15]. Diabetic patients suffer from multiple types of diabetes distress, including emotional distress, physician-related distress, interpersonal distress, and regimen-related distress. Diabetic depression is also related to genetic (family background history) and social factors to diabetes distress, which present the burden of disease and its treatment [16,17]. Recent studies have found that most diabetic patients with elevated depression are not clinically ill but suffer from diabetes-related distress [9,10,18,19].

In various studies [16,20,21,22], different countries were outlined regarding the prevalence of diabetes depression that varies from 18% to 35%. Around 17.23% of diabetic patients without diabetes depression were shown to have high diabetes distress in the subsequent 18 months of their initial assessment. It is not only burdensome but may also hamper the self-care of the patient and therefore affect glycemic regulation compared with nondepressed and nondistressed glycemia. Thriving community research has found that growing diabetes-related depression is connected to declining glucose metabolism [5,23]. It was also correlated with a poor evaluation of insulin care, and insulin-treated patients reported slightly higher diabetes pain relative to oral- or diet-treated patients. For example previous literature [24,25,26] strongly indicates that high levels of diabetes distress are related significantly to poor self-care, low diabetes immune effectiveness, and poor quality of life, even after psychiatric depression control.

The prominent aim of this work is to assess the depression, diabetes-specific distress, and demographic and sociographic influences in type 2 diabetes mellitus (T2DM) patients. Diabetes mellitus is an exponentially evolving disease in developing countries, e.g., Nigeria. The IDF cited a huge number of deaths associated with diabetes in the literature. The findings in [27,28,29,30,31] determine the factors associated with the prevalence of diabetes in Nigeria for primary health education; however, for the factors of distress and depression among Nigerian diabetic patients, this study is novel. It motivates us with significance to the clinical implications. Further, this study is significant because it has considered several sociocultural and religious differences between Nigeria and large undeveloped populated countries with low socioeconomic status and excessive epidemiological risk.

## 2. Materials and Methods

### 2.1. Ethical Consents

In compliance with the Nigerian Hospital Ethics Committees, this cross-sectional analysis study was accepted from where the data were gathered to execute a research paradigm with Reference Numbers MOH/SUB/4679/I, SMH/1580/V/IV, and MOH/ADM/744/1/553. All experiments and simulation procedures conformed to the Declaration of Helsinki. All participants provided written informed consent after having all procedures explained to them both in writing and verbally.

### 2.2. Inclusion and Exclusion Criteria

The eligibility requirements for this research were T2DM patients, including males and females, within the age bracket of 20 to 86 years. Furthermore, exclusion requirements included patients that needed emergency hospitalization and long-term treatments for other chronic diseases other than diabetes. Additionally, the analysis also omitted opioid-using patients and patients who failed to provide their full details.

### 2.3. Data Collection and Explanation

Data on 790 real-life T2DM patients were collected for the time frame of 2017 to 2020 via a questionnaire and scale measurements by the Beck Depression Inventory (BDI) [32], Diagnosis Statistical Manual (DSM) [22,33], and Diabetes Distress Screening (DDS) [20,34] from three major state hospitals throughout Nigeria and carefully checked. Seventy-eight patients were omitted out of 790 total patients based on the inclusion and exclusion criterion, and 712 patients were analyzed further for this study. The tributaries, namely with the percentage of patients suffering from T2DM, are Kebbi State Hospital (226, 31.74%), Sokoto State Hospital (296, 41.57%), and Kaduna State Hospital (190, 26.69%). In comparison, 368 (51.68%) were males and 344 (48.31%) were females out of 712 tested patients, as shown in Table 1 with their evaluation of age distribution.

### 2.4. Measurements

Initially, the BDI was used to evaluate and diagnose the disorder in patients (for both men and women) with T2DM. The BDI process consists of twenty-one groups: sadness, pessimism, guilty feelings, past failure, loss of pleasure, self-criticalness, suicidal thoughts, crying feelings, agitation, loss of interest, punishment feelings, self dislikes, indecisiveness, worthlessness, loss of energy, changes in sleeping patterns, irritability, changes in appetite, concentration difficulty, tiredness and fatigue, and loss of interest in sex. These inventories have four stages starting from 0 to 3, where 0 is considered as No, 1 is Weak, 2 is Mild, and 3 is Severe. The mean BDI score was used for the tests of all patients. The overall performance was mild (if ≤16) and moderate (if between 17 and ≤30). The significant causes of depression were considered (if >30). The DSM criteria for individuals with a psychiatric disorder (for 2 weeks of sensation) were also evaluated. Further, the DDS scale of seventeen separate questions was analyzed to evaluate the real diabetes distress in the patients. The DDS-17 covers four patient-related subscales of distress: emotional burden, physician-related distress, regimen-related distress, and interpersonal distress. Out of 17, the DDS scale was ranked from 1 (no distress from diabetes) to 6 (serious distress from diabetes). Table 2 provides the particulars of an average DDS scale ranking. The moderate level of distress (from 2.0 to 2.9) has been recognized as clinically significant.

### 2.5. Attributes and Statistical Analysis

Five attributes were chosen for the associated factor analysis, namely age, gender (male or female), physical exercise (yes or no), history of diabetes (yes or no), and body mass index (BMI). BMI was measured as body weight, separated by height squared into meters, and BMI (≥25) was defined as overweight. These five attributes were taken into account from our previously reported prevalence research [23,27,28,29,35,36]. Furthermore, the characteristics of smoking (yes or no) were applied to this study because smoking has been a significant factor in human depression or distress in clinical trials and psychiatric expertise [37]. Linear regression was utilized further to measure the forecast prevalence of depression over distress in type 2 patients of diabetes mellitus. The Social Science Methodological kit (SPSS-v.27 available at https://www.ibm.com/support/pages/downloading-ibm-spss-statistics-27 accessed on 2 April 2021) was used [38]. Continuous and group variables’ mean ± standard deviation, and frequencies and percentages were calculated. The Chi-square test was used for categorical-scale parameter definitions of two or more categories [39]. Significance was measured at 5%.

## 3. Results

A total of 712 (*n*) patients, including 368 (51.68%) males and 344 (47.31%) females, were considered and examined with type 2 diabetes mellitus. Patient age and BMI mean were calculated as 48.6 years ± 12.8 and 45.6 years ± 8.3, respectively, for this study. Moreover, Table 3 demonstrates the assessment evaluation of BDI and DDS. Out of 712 patients, 640 (89.89%) were graded “positive” for depression, and the remaining 72 (10.11%) were screened as having no depression. From 89.89%, 14.37% deemed to be less depressed, 43.75% mild, and 41.87% extremely depressed. The prevalence of depression was 22.06%, and 638 (51.83%) patients were rated as having depression according to the DSM manual, both males and females. The prevalence was also counted as 24.08% for DDS assessments of specific distressed patients. In 712 patients, 628 (88.20%) were counted as distressed, including 28.96% who were deemed distressed by mental- and emotion-related distress, 16.42% by medical-related and physician-related distress, 13.21% by regimen-related distress, and 23.61% by interpersonal-related distress and depression.

The remaining 84 (11.79%) were counted as less distressed. Moreover, Table 4 shows the relationship of type 2 diabetes mellitus (distressed and depressed) patients based on BDI and DDS assessments in nondepressed (72 (10.11%)) patients according to the parameters of DSM manual by containing the confidence interval of 95% with *p* < 0.001. Recent literature shows that five characteristics, including marital status, BMI, occupation, education, and income, are not significant with diabetes mellitus depression and distress, according to the univariates Chi-square analysis. This research does, however, involve characteristics such as age, sex, physical fitness, diabetes background history, and smoking that shows significance in Table 5 with diabetes mellitus depression and distress according to the multivariate regression analysis with confidence interval 95% and *p* < 0.001.

**Figure 1:**
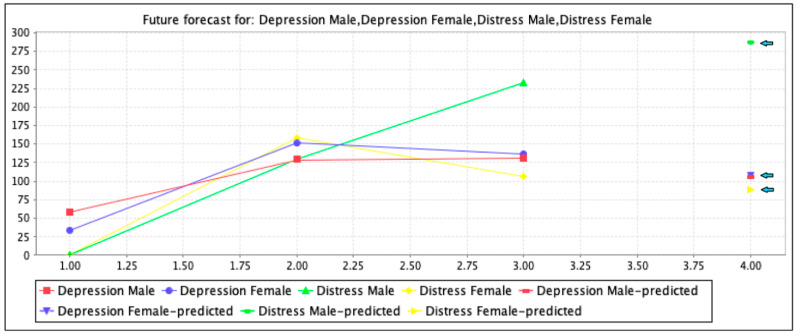
Forecast prediction of the analyzed population.

Figure 1 shows the prediction analysis of depression and distress present separately from the utilized population in this study according to Table 3. Vertically is shown the total number of male and female patients with depression present in males (little = 58, moderate = 129, and severe = 131) and females (little = 34, moderate = 151, and severe = 137), as well as distress present in males (moderate = 130 and severe = 233) and females (moderate = 158 and severe = 107). An analysis of Table 3 showed that mild distress was measured as 0 in both males and females. Moreover, horizontally is shown the level frame forecast prediction analysis by linear regression with a confidence interval of 0.95%. The future forecast prediction results of Figure 1 indicate that in the population of all 710 T2DM patients (by considering the utilized attributes), the male depression population can be considered 108 and female 112. Further, the distressed male population can be predicted as 283 and the female population as 90. The prediction points are marked with a black-colored arrow for more visibility. These forecasting analyses were measured by the linear regression M5 method considering the confidence interval (CI) of 0.95%.

**Figure 2:**
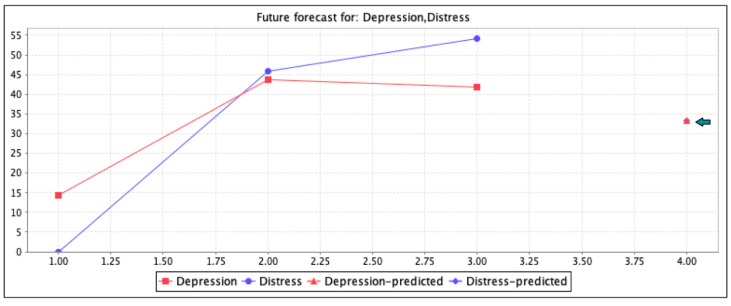
Forecast analysis of depression over distress.

Figure 2 shows the prediction forecast analysis of depression over distress in utilized T2DM patients according to Table 3. Vertically is shown the total percentage population of male and females patients with depression present (little = 14.37%, moderate = 43.75%, and severe = 41.87%) and distress present (moderate = 45.86% and severe = 54.14%). In Table 3, the mild distress was measured 0 in both males and females. In addition, horizontally is shown the level frame forecast prediction analysis of depression over distress (33.33%) with a confidence interval of 0.95%. The prediction points are marked with a black-colored arrow for more visibility. These forecasting analyses were measured by the linear regression M5 method considering the confidence interval (CI) of 0.95%.

## 4. Discussion

The present research study identifies and determines the status and related factors of diabetes distress and depression. In this study, the most fundamental and main findings of the prevalence ratio were 22.06% in depressed patients and 24.08% in distressed patients of type 2 diabetes. The ratio is much higher than the previous and most recent research, as shown in Table 6. Variations in prevalence ratios have been reported in previous studies owing to the inclusion and exclusion criteria for patients, the data collection, and the screening process. According to our best interpretation, this research report is the first in Africa (specifically in Nigeria) to be undertaken in patients with type 2 diabetes with the prevalence of depression and distress, along with relevant causes and characteristics. The findings indicate that a significant percentage of the population in Nigeria suffers from diabetes mellitus and depression, based on the data collection. The research also evaluates that females are more depressed and anxious than males. This might involve breastfeeding, menstruation periods, lack of sleep, lack of exercise, and other factors, such as employment, friends, homes, and so on [40,41,42]. Bener et al. [22] and Fisher et al. [20,21] concentrated on the greater proportion of women with depression. In contrast, emotional-based distress was found to be raised in the surveyed population by 28.96% compared to interpersonal-related distress, followed by physician-related distress and regimen-related distress of 23.61%, 16.42%, and 13.21%. This prevalence ratio can be an aspect of disease self-management. This research identified smoking, physical fitness, and history of diabetes to be the main indicators of depression and distress. The most commonly used methods for depression appraisal were: Patient Health Questionnaire (PHQ) [43,44,45], Manual for Depression Epidemic Study Center (CESD) [46], and the BDI. However, there are already major variations in different studies [14,33,47,48,49,50]. The key importance of this research was to determine patients who were considered to not be stressed but have diabetes distress according to the DSM manual guidelines. Furthermore, Roy et al. [51] claimed that BDI or other self-reporting instruments could only be used to identify signs that could then contribute to clinical interviews. Simultaneously, misidentifying distressed patients with diabetes as having depression may contribute to excessive antidepression prescription. Zhang et al. [52] further confirmed the theory that even low levels of depression may have a major detrimental influence on a commitment to medication and also proposed that diabetic distress is an important predictor of mild depression that could impede adhesion and self-managing of the care. They proposed a tailored clinical technique, for instance, utilizing both diabetes distress and depression scale, which would be an important instrument for reducing psychological issues and encouraging attention to medication in type 2 diabetes mellitus patients.

### Strength and Limitation

The analysis does not have any limitations; however, genetic susceptibility may play a role in determining the prevalence of diabetes-specific distress and depression that may also be further investigated. In addition, this study is significant because it considered several sociocultural and religious differences between Nigeria and large undeveloped populated countries with low socioeconomic status and excessive epidemiological risk. However, some clinical objective methods can be further implemented to cover more demographic and sociological features for screening and diagnostic studies.

## 5. Conclusions

Type 2 diabetes mellitus patients seem to be susceptible to depressive distress. In this research, distress and depression were prevalent in Nigerian patients with type 2 diabetes mellitus. The findings reveal that the main predictors were age, sex, the history of diabetics, fitness, and smoking. Low detection rates are a major impediment to successful diabetes management that requires early diabetic evaluation and distress treatment.

This study includes 790 diabetes patients between 2017 and 2020, including factors for analyses such as diabetic distress, the Beck Depression Inventory, diplomas, and Chi-square findings. Moreover, several sociocultural and religious differences between Nigeria and the large undeveloped populated nations with low socioeconomic status and an excess of epidemiological risk have been considered during the experimentation of this work.

Consequently, it can be expected that the problems of distress must be studied and addressed in therapeutic environments. Causal depression and distress are a key goal of potential research in patients with type 2 diabetes. Further, the clinical consequences of type 2 patients with diabetes mellitus for their early screenings are important.

## Figures and Tables

**Table 1:** Distribution by age of the current study population.

Hospital	Age Distribution	Male (*n* = 368)	Female (*n* = 344)	Total (*n* = 712)
Kebbi	≥20 to <40	30 (8.15%)	51 (14.83%)	226 (31.74%)
	≥40 to <60	49 (13.31%)	30 (8.72%)	
	≥60 to ≤86	37 (10.05%)	29 (8.43%)	
Sokoto	≥20 to <40	23 (6.25%)	46 (13.73%)	296 (41.57%)
	≥40 to <60	61 (16.57%)	61 (17.73%)	
	≥60 to ≤86	53 (14.40%)	52 (15.12%)	
Kaduna	≥20 to <40	45 (12.23%)	24 (6.98%)	190 (26.69%)
	≥40 to <60	34 (9.24%)	22 (6.39%)	
	≥60 to ≤86	36 (9.79%)	29 (8.43%)	

**Table 2:** Diabetes-specific distress subscale items break-down.

Distress Subscale	Average Items	Items Break Down	Mean Score
Emotional Burden	5	1, 4, 7, 10, 14	▪ If <2.0 then no distress ▪ If between 2.0 to 2.9 then moderate distress ▪ If ≥3.0 then severe distress
Regimen distress	5	3, 6, 8, 12, 16	
Physician distress	4	2, 5, 11, 15	
Interpersonal distress	3	9, 13, 17	

**Table 3:** Presence measures of diabetes distress and depression in the study population.

Patients (*n* = 712)	Depression Present (*n* = 640, 89.89%)			Distress Present (*n* = 628, 88.20%)	
	Little	Moderate	Severe	Moderate	Severe
Male	58 (9.06%)	129 (20.16%)	131 (20.47%)	130 (20.70%)	233 (37.10%)
Female	34 (5.31%)	151 (23.59%)	137 (21.40%)	158 (25.16%)	107 (17.04%)
Total	92 (14.37%)	280 (43.75%)	268 (41.87%)	288 (45.86%)	340 (54.14%)

**Table 4:** Diabetes distress and depression relationship based on the Beck Depression Inventory (BDI) and Diabetes Statistics Manual (DSM) in nondepressed patients.

Distress	Depression Absent	Depression Present	Odd Ratio (95% CI)
Absent	18	0	1.61 (1.34–2.96)
Present	40	12	

**Table 5:** Regression assessment of significantly associated predictors.

Associated Attributes	Total (*n* = 712)	Multivariate Assessment	
		Distress OR	Depression OR
Age		2.8 (1.4–4.9)	3.3 (1.1–5.8)
≥20 to <40	219 (30.76%)		
≥40 to <60	257 (36.09%)		
≥60 to ≤86	236 (33.15%)		
Gender		4.8 (2.9–6.9)	4.9 (2.7–7.1)
Male	368 (51.68%)		
Female	344 (48.31%)		
Smoking		3.5 (2.1–4.3)	3.3 (2.1–4.1)
Yes	328 (46.07%)		
No	384 (53.93%)		
Diabetes history		3.7 (1.7–6.7)	4.2 (2.2–6.2)
≤5 years	517 (72.61%)		
>5 years	195 (27.38%)		
Physical exercise		3.3 (1.3–6.3)	3.9 (1.5–6.6)
Yes	277 (38.90%)		
No	435 (61.09%)		

**Table 6:** Prevalence comparison of the current study with previous studies.

Countries	Prevalence Ratio		References
	Distress	Depression	
Nigeria	24.08%	22.06%	Current study
Saudi Arabia	23.03%	20%	[14]
Australia	7%	6.02%	[2,40,53,54,55]
Germany	8.09%	7.04%	[2,34,40,53,54]
India	18%	17%	[33,49]
Spain	18.06%	20%	[2,40,53,54,56]
Canada	23%	12%	[2,40,53,54,57]
Pakistan	20.05%	14.07%	[2,40,53,54,58]
Iran	21.04%	18.04%	[2,40,48,53,54]

## Data Availability

Real-life type 2 diabetes mellitus data collected only from Nigeria used in this study are available on a reasonable request.
